# Virtual reality (VR) as a simulation modality for technical skills acquisition

**DOI:** 10.1016/j.amsu.2021.102945

**Published:** 2021-10-27

**Authors:** Aussama K. Nassar, Farris Al-Manaseer, Lisa M. Knowlton, Faiz Tuma

**Affiliations:** a Stanford University School of Medicine, Stanford, USA; b Central Michigan University College of Medicine, Saginaw, USA; c Chicago Medical School, Medical Student year 3, USA

**Keywords:** Surgical education, Simulation, Virtual reality, Surgical skills, Educational technology

## Abstract

Efforts continue to facilitate surgical skills training and provide accessible and safe training opportunities. Educational technology has played an essential role in minimizing the challenges facing traditional surgical training and providing feasible training opportunities. Simulation and virtual reality (VR) offer an important innovative training approach to enhance and supplement both technical and non-technical skills acquisition and overcome the many training challenges facing surgical training programs. To maximize the effectiveness of simulation modalities, an in-depth understanding of the cognitive learning theory is necessary. Knowing the stages and mental processes of skills acquisition when integrated with simulation applications can help trainees achieve maximal learning outcomes. This article aims to review important literature related to VR effectiveness and discuss the leading theories of technical skills acquisition related to VR simulation technologies.

## Highlights

• VR simulation offers an innovative training approach to supplement both technical and non-technical skills acquisition.
• VR simulation with haptic feedback is a promising modality for safe, repetitive, and learner-oriented operative training.
• VR simulation facilitates deliberate practice with built-in auto feedback to address limited staff resources.
• To maximize the effectiveness of simulation, an in-depth understanding of the cognitive learning theory is necessary.

## 1 Introduction

Training on advanced surgical skills continues to be a challenge for trainees, instructors, and residency programs. Among surgical specialties, technical skills acquisition requires extensive psychomotor skills training. In recent years, training objectives have shifted toward competency-based medical education (CBME), focusing on outcomes [1], which mandate sufficient training to achieve the required competencies. With the several current limitations, achieving competency in psychomotor skills can be significantly challenging to surgical programs. In addition to the everyday challenges of limited operative room (OR) access and time, patient safety concerns, and 80-h work limit, there are extra challenges that include access to simulation labs and training opportunities, as well as the availability of qualified mentors [2].

Educational technology applications such as simulation offer alternative training opportunities outside the operating room without putting patients at risk. Simulation has been proposed as a learner-centered and safe approach to technical skills acquisition with ample opportunity for repeated, safe practice [3]. It is also known for its ability to replicate rare and risky clinical scenarios in a controlled environment. With simulation, educators can create various learning experiences tailored to the educational needs of the trainees at all levels of training [4]. Norman et al. demonstrated in their review that simulation-based education was associated with consistent improvement in skill acquisition compared to traditional control groups [5]. However, there was a questionable advantage of high-fidelity over low-fidelity simulation.

Simulation can be classified into physical, virtual reality (VR) and a hybrid model [3]. VR is the focus of this review. This article will review the effectiveness of VR, analyze the role and integration of VR in surgical education in light of technical skills acquisition theories, and critique VR as a simulation modality. Based on the review, recommendations and conclusions will be advocated for use by educators, programs, and institutions.

### 1.1 Virtual Reality

Webster's online dictionary defines VR as "an artificial environment which is experienced through sensory stimuli (as sights and sounds) provided by a computer and in which one's actions partially determine what happens in the environment; *also*: the technology used to create or access a virtual reality." The first known use for the term Virtual Reality was in 1987, where it referred to the use of head-mounted devices (HMD's) by fighter pilots [6]. Since that time, its application has expanded into aviation, police, military, and medicine.

Virtual reality, in technical terms is used to describe a three-dimensional, computer-generated environment, which can be explored and interacted with by a person. That person becomes part of this virtual world or is immersed within this environment, and while there, can manipulate objects or perform a series of actions [6]. Augmented reality (AR) is another term used interchangeably with VR. True virtual reality completely blocks out the real world, whereas augmented reality adds to the already existing real world [7].

To review and evaluate VR as a simulation modality for skill acquisition, it is helpful to describe it in the context of a real-life example. The best illustration occurs in training a novice junior resident in laparoscopic cholecystectomy (removal of the gall bladder utilizing minimally invasive surgery), a standard procedure considered by surgeons as the benchmark of early laparoscopic skill acquisition. For this review, virtual reality will be the modality proposed to develop the necessary skills needed to perform this procedure, adhering to the fundamentals of information processing theory. The ultimate goal will be to transfer the laparoscopic skills learned using a solely VR-based simulation modality to the operating room with safety and competency in a supervised setting. Virtual reality, like any other simulation modality, is designed to develop simple and complex motor skills. Fitt's and Posner's three-stage model of motor learning is widely accepted in both the surgical literature and motor skills literature and thus will be used as a guide throughout our critique of the effectiveness of VR [8,9]. An appropriate review of the literature will support this review.

### 1.2 Skill acquisition

The term skill in this article refers to the accurate and coordinated perceptual-motor performance and not to skills as that might be applied to non-motor tasks [10]. The motor program is a representation of an action that is centrally stored, comprised of the cognitive and motor elements of movements, as well as the specifications for carrying out those movements [11]. Until recently, it has been impossible to look inside the brain's central nervous system to see how motor learning occurs. Thus, two theoretical perspectives of motor learning emerged. The first is the information-processing theory rooted from experimental/cognitive psychology. The second is the ecological approach that emerged from ecological psychology and dynamical systems theory [12]. In the literature, these two theories are not considered to complement one another, nor do they enhance each other's characteristics [10].

**Fig. 1:**
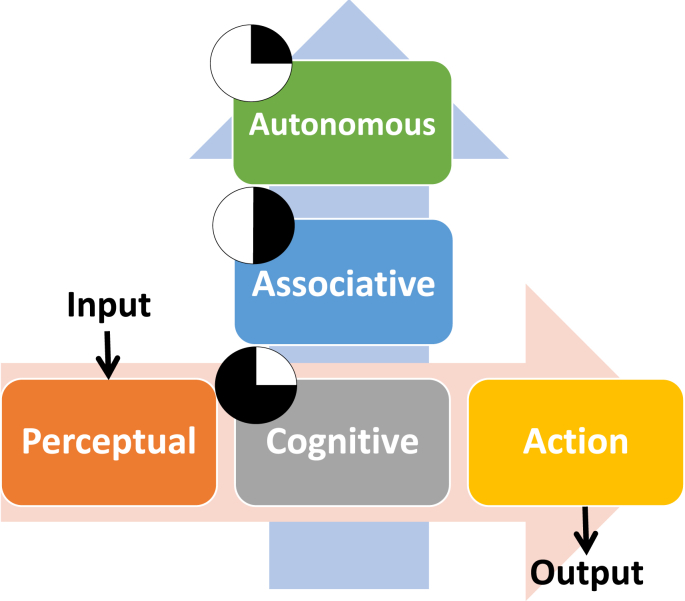
Fitts–Posner Three-Stage Theory of Motor Skill Acquisition conceptual framework.

Information processing theory was developed to examine measures of behavior and make conclusions or inferences on how learning occurs inside the brain. This is based on models that depict the flow of information in humans similar to a computer system; thus, the approach is mechanistic. The brain works in a set of sequences similar to a computer [10]. The assumption of three basic information processing subsystems; perceptual input, process the information, and output through actions (Fig. 1) [10].

The Information processing theory itself is comprised of two main theories: the closed-loop [12] and schema [11] theories. The leading theory proposed within the ecological approach is the theory of constraints [13]. Both closed loop and schema theories were frequently criticized for their inability to serve as general theories of coordination, control, and skill. However, this lack of generalization might have had more to do with the specific nature of each theory and less to do with the information processing approach per se [10].

The three stages of motor learning are described by Fitt's and Posner's three-stage Model of Motor Learning in order of progression, and they are the cognitive, associative and autonomous stages (Fig. 1) [14].

### 1.3 Cognitive stage

The cognitive stage of skill acquisition is characterized by the early identification and understanding of the skill to be learned involves very little motor skill. During this initial stage, explanations of the relevant surgical anatomy, demonstration of the procedure, and the use of instruments with tissue handling and energy sources applications are processed in this stage. The trainee intellectualizes the task, and his performance is erratic and with distinct steps (Reznick et al., 2006) [8]. During this stage, the learner is more cognitively overloaded with the least free working memory (Fig. 1).

For centuries, human cadaver dissection has traditionally been considered the gold standard for 3D spatial understanding of humans' surgical anatomy. This understanding was not attainable via teaching using a 2D textbook. However, the traditional teaching of anatomy by dissection is declining and is not without disadvantages, mainly due to difficulties in feasibility and portability [15]. Another method is the use of a well-made simulated plastic model. However, this method has disadvantages, including considerable cost and portability limitations. Virtual reality is an alternative way to present three-dimensional anatomy [16]. Hence the application of VR in teaching and assisting in developing the cognitive aspect of the background knowledge for the procedure has been used. The advances in educational technology created a plethora of 3D surgical anatomy software applications. These software applications are now carried in most trainees and surgeons' pockets as a quick reference before surgery. Hence, mobile learning established rapid inroads into the provision of surgical education [17].

In reference to the laparoscopic cholecystectomy example, the novice trainee must learn the relevant surgical anatomy and the principles with the main steps of the procedure to plan and execute the surgical approach of laparoscopic cholecystectomy properly. Using an exclusive VR simulation-based model of learning, the trainee must review the relevant anatomy on 3D based anatomy software, with the ability to interact and manipulate the model in multiple views to conceptualize the surgical approach of the procedure.

An interesting study by Garg et al. concluded that the dynamic display of multiple orientations offered only a minimal advantage to some learners, and stated that it might disadvantage learners with more inferior spatial ability [18]. A concern with this study's outcome is that it involved 87 first-year medical students and was conducted during the orientation week in 2 h. They also chose one of the most complex bony structures of the human body with no power calculation. These results were also consistent with two other studies conducted by Stepan et al. which analyzed short-term and long-term memory retention and Shao et al. which observed student learning of basic theory, clinical manifestations, anatomy, and surgical methods [19,20].In Stepan et al., a mixture of 64 first-year and second-year medical students participated in a randomized study comparing virtual reality neuroanatomy to online textbooks [19]. There was little difference in the scores of both groups in short- and long-term retention. However, the VR group showed higher motivation levels when learning the material. Their overall experience was significantly positive and more engaging. Thus, the tests were performed with novice learners for whom even minor stimuli might create a relatively high cognitive load. Shao et al. followed a subjective questionnaire and scored assessment to evaluate the effectiveness of VR. Students performed significantly better in the following categories of basic theory, tumor location, adjacent structures, clinical manifestation, and diagnosis [20]. The results followed along with a more recent study in which Blumstein et al. observed two groups of medical students in a standard and virtual group in learning intramedullary nailing [21]. 20 students were split evenly and observed regarding their development of procedure time and motion, instrument handling, knowledge of instruments, flow of operation, and knowledge of procedure. The VR group scored statistically higher in both short term and long term retention of skills than their standard counterparts [21]. The extent to which findings would be applicable for advance or experienced learners is evaluated in Lohre et al. [22] 18 senior surgical residents were randomly placed into an immersive virtual reality group and a control group. The VR group yielded a higher objective structured assessment of technical skills and showed a higher transfer effectiveness ratio. Both novice and experienced learners benefitted substantially from the VR simulations [[19], [20], [21], [22]]. Future research should be conducted to further explore the benefits in experienced individuals.

Hopkins et al. found that virtual models made no significant difference in anatomy knowledge before or after-test for undergraduate students [23]. The researchers observed that students positioned the model according to mirror images in the textbook without fully exploring all of the options for repositioning the model. It may be that for the novice learner, their stage of learning means that they were not able to leverage the potential for manipulating the 3D model.

Yoganathan et al. studied virtual reality application for acquisition of knot tying skills. In total, 40 first-year post-graduate residents were randomly categorized into either a 2D video teaching or a 3D immersive model [24]. The study concluded that the knot tying scores were significantly improved in the VR teaching group with a knot score of 5.0 compared to their 2D counterparts who received an average knot score of 4.0. The scores were assessed by an individual competent in knot tying.

In a study performed by Pulijala et al. 95 post-graduate residents from years one to three were assigned to a VR group or non-VR group to develop the skills to perform a Le Fort I osteotomy [25]. The confidence and knowledge of the VR group improved significantly, with the most significant change being in the first-year post-graduate residents. It is important to note that the most significant changes were in advanced trainees who were still in their early stages of training, suggesting a possible median for success.

It is the authors' experience that novice trainees are cognitively overloaded with the amount and complexity of new information involved in learning surgical anatomy, with a relative decrease in free working memory. Hence, they resort to the simplicity of a single view, i.e., the textbook view, versus exploring more in-depth nuances that could be offered by 3D VR. The advanced trainees who have already mastered appreciation for basic anatomical relationships will focus more on the unusual and varied views to enhance their 3D perception of a particular body part. This translates to the ability to perceive the gall bladder's relevant critical structures from multiple views when the gall bladder is retracted and manipulated by instruments from different angles in the actual procedure.

### 1.4 Associative stage

During the second stage, the association stage, information from the cognitive stage, is used to associate specific stimuli with the relative response. This stage is characterized by increased speed and less error of less than 1% [10]. The associative stage is the stage of deliberate practice and feedback with more fluid movements and less interruption [8]. For our laparoscopic cholecystectomy example, it is best broken down into discreet tasks. Here, the resident learns precision placement tasks. The VR system used in this example is the LapSim Haptic System from SurgicalScience using the LapSim Basic module. It offers 13 different exercises relevant to our proposed laparoscopic cholecystectomy (camera and instrument navigation, coordination, grasping, cutting, clip applying, lifting and grasping, suturing, precision and speed, fine dissection, seal and cut). This practice is best done as part training in a high contextual interference (random) fashion and distributive practice [26,27]. After conducting these basic procedures, the trainee will practice on the LapSim Haptic System and work on the proposed surgical procedure, laparoscopic cholecystectomy, as a whole practice. As mentioned earlier, the associative stage is composed of deliberate practice and feedback. At this stage, the trainee has less cognitive overload and is able to free more working memory (Fig. 1).

**Deliberate practice**. Ericsson introduced the concept of deliberate practice "as a highly structured activity, the explicit goal of which is to improve performance. Specific tasks are invented to overcome weaknesses, and performance is carefully monitored to provide cues for ways to improve it further." [28] In the laparoscopic cholecystectomy skill acquisition example proposed here, deliberate practice is done via a VR simulation modality with the touch of a button, and with no additional materials and resources required in comparison with other simulation modalities, e.g., laparoscopic box simulator, which requires sutures, endoloops, etc. The drawback of VR is the fixed number of programmed scenarios stored for the laparoscopic cholecystectomy. The problem with this drawback is that the trainee will not be challenged anymore after several practice sessions.

In the haptic intrinsic feedback system, the perceptual experience was limited to the most fundamental basics. However, in more recent developments, haptic feedback has improved to properly incorporate tactile and kinesthetic feedback through a mechanism known as haptic rendering. Haptic rendering uses a polyhedral model that evaluates the force lines to create a more realistic feel for VR stimulation. The recent improvements can allow individual medical students to feel what suturing a blood vessel feels like in anastomosis [29]. Despite prior weaknesses of VR, there is evidence of its effectiveness in the surgical context, specifically with laparoscopic cholecystectomy. An RCT randomized novice surgical residents to a deliberate practice (DP) on a VR group and a conventional residency training group [30]. Both groups performed Laparoscopic cholecystectomy in the OR afterward. The DP group had a superior technical performance post-intervention at *P* = 0.03. Palter et al. concluded that the DP group on a VR simulator improves technical performance in the OR.

Another procedure involving placement of pedicle screw in a spine surgery simulation was assessed by Gasco et al. in an RCT consisting of 26 senior medical students [31]. The students were randomized to a VR simulation and non-simulation with a traditional visual/verbal instruction group and assessed after a single session of training. A total of 52 pedicle screws were analyzed, with the average number of errors per screw being 0.96 for the simulation group as opposed to 2.08 in the non-simulation group. In their study, the most significant reduction in errors involved the coronal, length, and pedicle breach categories – most likely due to the ability to develop a mental map of the anatomy due to the 3D images presented.

Other RCTs evaluated the efficacy of VR training compared to other simulation modalities. In an RCT that observed the development of basic laparoscopic skills in both a box trainer and a VR trainer, 36 medical students randomized into the two groups [32]. The study used the Global Operative Assessment of Laparoscopic Skills (GOALS) score to rank depth perception, bimanual dexterity, efficiency, tissue handling, and autonomy with a scoring system of 5 points for each category. The results show a higher score for the box-trained group at 15.31 ± 3.61 compared to their VR counterparts at 12.92 ± 3.06; however, both groups showed significant improvement in acquired laparoscopic skills.

Another RCT observed the development of arthroscopic skills in a comparison of VR learning to benchtop learning among 17 medical students and interns [33]. The study was evaluated primarily on performance based on the motion analysis using the Wireless Assessment of Surgical Performance (WASP) to compare the total time taken and the number of hand movements. Both groups showed statistically significant improvement in total time taken (178.8 s less for benchtop and 67 s less for VR) and hand movements (177 fewer hand movements for benchtop compared to 74.5 fewer hand movements for VR) with the benchtop group showing a greater difference overall.

Banaszek et al. conducted a study using a global rating scale as a primary outcome to evaluate the benefits of VR versus benchtop simulation [34]. There were 40 medical students separated into three different groups – a VR simulator, a benchtop simulator, and a control group. Based on the global rating scale, the VR group performed considerably higher than the benchtop group and even higher than the control group in both practice and skill transfer. In a review from. McKnight et al., three studies were evaluated for knowledge development in a high fidelity visual-haptic feedback simulator through the global rating scale and procedural checklist [35]. The studied inferred training with a haptic simulator indicated a greater understanding of surgical procedure with one study yielding a significant improvement of the global rating scale.

**Feedback**. Feedback refers to specific information trainees receive about their technique to improve future performance [36]. Feedback has been shown to have motivational properties in addition to information that prompts learning [37]. Feedback has obtained special attention in medical education due to the growing role in education [38]. Feedback can be classified as augmented or intrinsic. Augmented feedback given by an instructor either involves information about the outcomes of movements (Knowledge of Results) or the quality of movement pattern, aiming to correct improper techniques (Knowledge of Performance). A good example of augmented feedback is watching a video after the end of a performance to be aware of incorrect movements and patterns [39]. Intrinsic feedback refers to the actual sensory-perceptual experience that the trainee experience as a natural part of performing a skill, such as the haptics, the audiovisual etc. [40].

VR simulation systems usually have a built-in objective evaluation for trainees' performance given at the end of each procedure. The feedback includes a pass or fail mark and a comprehensive breakdown of scores achieved for outcomes such as time, bleeding, the volume of bleeding, injuring essential structures, which rank the trainee with her/his peers. Snyder et al. randomized medical students to either proctored training (automated simulator feedback plus human expert feedback) or independent practice (simulator feedback alone) [41]. After proficiency on the VR, they were taken to a live porcine model to assess skill transfer. The study concluded that proctored VR training was no more effective than independent training with respect to performance.

The feedback of the VR system was analyzed for consistency and accuracy by Wijewickrema et al. [42] A randomized control trial in which the performance of 24 medical students was evaluated based on drill performance for a virtual cortical mastoidectomy. The students were randomized into feedback or non-feedback group. The results of the study show that students yielded consistent improvement with a positive interaction with the VR system. The feedback was then analyzed based on whether it was a false-positive, wrong feedback, and false-negative. This yielded accurate feedback provided in 84.2% of the cases.

### 1.5 Autonomous stage

In the autonomous stage, cognition is less of a factor, and movement becomes automatic with high consistency and little error - approaching 0% [10]. This stage is also characterized by the least cognitive overload and with the highest available working memory (Fig. 1). The less cognitive overload results in decreased stress and decreased interference of other activities [43]. Could the VR simulator help our residents reach the autonomous stage faster for the proposed laparoscopic cholecystectomy procedure? This could be achievable with deliberate practice; the trainee might reach this stage on the SimLap scenarios. But the more critical issue is how this would transfer to a real-life application in the operative room setting.

### 1.6 The impact of VR on surgical training

Findings from studies to date relating to the impact of VR simulation on learning outcomes at the various stages of skill acquisition were mixed. The available results in the literature are reviewed and discussed by outcomes in summary below. The authors selected the most relevant and applicable studies for this discussion based on the principles of surgical education.

A well-executed RCT by Larsen et al. compared 6 h of LapSim VR training for 24 novice OBGYN trainees to a control group [44]. The VR intervention group showed superior skill transfer in the OR and had significantly decreased operating time compared to the control group. As a consequence of this study, Denmark announced national standards requiring laparoscopic training on VR for all OB/GYN residents before certification. The widespread modification of training requirements is a significant reflection of the impact of this study on surgical skills training. Another RCT done by Calatayud et al. showed that a 15-min warm-up using LapSim VR prior to performing laparoscopic cholecystectomy by surgeons showed improved OR performance [45]. The aspect of improvement in this study needs further description and characterization. The practicality of performing "warming-up" in real-life practice is questionable, given the fast pace surgery environment.

An RCT Logishetty et al. assessed the skill transfer of a total hip arthroplasty (THA) among 24 surgical trainees [46]. A control group of 12 trainees received conventional preparation while the VR group received six weeks of VR preparation. The trainees were then assessed for their ability to perform a cadaveric THA. The results yielded that the VR-trained trainees showed more key steps, were faster and used orientated tools better than the control group.

Three other high-quality studies [34,47,48] found that 3D VR offered significant advantages over 2D (mainly for novices) including faster times in surgery for both novices and advanced users [[48], [49], [50], [51]], improved spatial ability for novices [52], improvements in executing skills for novices doing a nerve hook task [53] and for novices attempting 5/6 laparoscopic surgical tasks (but not for residents attempting the same task) [54], and reduced error rates for novices [48]. The Banaszek et al. study conducted a transfer of skill aspect to their study and observed that the VR group showed a higher ability to transfer their skills in both a crossover test on the benchtop stimulation and a cadaveric experiment [34]. Based on these findings, it appears that 3D VR offers some advantage compared to conventional training for error rates for novices, some skill execution advantages for novices (which may be context-specific), and faster times for both novices and advanced learners.

Two well performed studies evaluated the translation of cognitive load and disruptions in virtual reality to improve performance of surgical outcomes and environment creation [55,56]. In Sankaranarayanan et al. the difference in handling secondary outcomes tied directly to experts already being in the autonomous stage [55]. The RCT conducted placed 11 novice medical students into either a control, VR, and a VR + cognitive load (CL) group. Performance was evaluated based on the students’ ability to perform prior to training, after training, and two weeks after completing their training. The primary task was a VR-based peg transfer with the secondary cognitive load task being a two-digit math multiplication. Both the VR and VR + CL performed significantly better in their post-test from their baselines and compared to the control groups. With the use of VR simulation, learners can be placed in an environment similar to that of an actual operating room without the risk. Well known negative predictors of poor surgical outcomes include auditory and visual distractions. In Krüger et al., 42 surgeons of different expertise and fields were asked to participate in a stress training unit [56]. The aim of the study was focused on the negative effects of auditory and visual distractions on outcomes. A non-optimal environment was created to expose physicians to what would be a seemingly plausible scenario such as loud devices and interactive disturbances.

On the other hand, several studies showed no significant advantage of 3D VR over traditional modalities in terms of skill or task completion or procedure times for a particular task/total procedure [18,33,[57], [58], [59], [60], [61], [62]]. Brewer et al. found no significant difference in the means of post-test scores for medical students, but the intervention consisted simply of the availability of 3D snapshots [57]. Dziegielewski's well-conducted study observed a more complex intervention of 3D biomodels for assisted reconstruction (after a brief 2-min practice) [60]. However, that intervention still led to plates with statistically-indifferent projection and splay compared to the control (p < 0.05) for both residents and surgeons (only some of whom were afforded minimal practice). This study described randomization and standard procedures as well as rigorous outcome assessment and thorough details for the task expected of participants; however, all of these criteria were not consistently met for most studies. Honeck's prospective cohort study did not find any difference in times for specific skills or total procedure time [58]. The Podolsky study provided the most training and practice at 1 h (total) and found that it did not result in improved screw placement for trainees (P = 0.34), or any differences between residents and surgeons (p = 0.37), or improved angulation of inserted screws (p = 0.34) [61]. The Middleton et al. study had individuals from both the benchtop simulator and the VR simulator to practice in the other group [33]. The benchtop group performed significantly better on the VR simulation as opposed to the VR group, who performed poorly on the benchtop simulation. In the Brinkmann et al. study, skill transfer was evaluated in a simulated laparoscopic cholecystectomy [32]. Both groups showed improvement in laparoscopic skills; however, the box-trained group performed higher in the procedural model. The literature on VR learning effectiveness was lacking in terms of overall study quality. More consistent use of more robust research designs is required to provide definitive answers as to whether and how VR works and in what particular group it will be more effective.

## 2 Conclusion

Surgical training in the last decade has been faced with several challenges in trying to achieve competency in surgical skills while being mindful of legal and ethical concerns with regards to patients' safety, staff work hour constraints, and limited OR resources. Technical skills training should commence outside the OR on a simulator. Supervised OR training is essential, but VR can be used to validate and fine-tune acquired skills to achieve proficiency and independent practice. VR simulation with haptic feedback is a promising training modality for achieving safe, repetitive, and learner-oriented operative training [62].

The major strengths of VR compared to other simulation modalities is that it facilitates deliberate practice with built-in auto feedback helping to address limited staff resources. VR requires no supplies and minor maintenance, is easily upgradeable, and offers one system that could be set up for multiple procedures and specialties. It is operator-friendly, accessible, portable, and easy to set up. The newer generations can be enhanced with haptic feedback. Its effectiveness for skill acquisition and transfer to the real-life operating room is supported by mixed evidence.

The challenges of VR compared to other simulation modalities vary from the mixed evidence for skill acquisition and transfer, potential high costs of the initial purchase. In addition to the lack of psychological fidelity, and the limited case practice scenarios. Its fidelity is defined according to the goals set to achieve. Rendering software responsible for a part of training may result in unpredictable exposures. Further, the heterogeneity in many studies by the nature of skill acquisition relative to environment has been difficult to assess which creates the need for further investigation [35].

VR has been gaining increasing applicability and acceptability across various surgical specialties. VR has potential strengths and weaknesses. Will VR become the future of surgical education? With the rapid acceleration at which computer technology is advancing, it will potentially be used as ubiquitously as in the aviation industry. With the prime advantage of broad versatility across several specialties and unlimited procedures store, VR is expected to be one of the leading simulation modalities of the future.

All authors approve the submission and disclose no financial conflict.

No funding was received for the study.

## Please state any sources of funding for your research

The authors report no funding

## Ethical approval

This a review study on education that involves no patient identifiers. Therefore, no ethical approval or consent were needed.

## Consent

This is a review study of education that involves no human beings. Therefore, no ethical approval or consent were needed.

## Author contribution

**Aussama K. Nassar MD, MSc** Conceptualization, writing original draft, editing &reviewing, visualization, project administration.

**Farris Al-Manaseer MSc w**riting original draft, editing.

**Lisa M. Knowlton MD, MPH** writing original draft, editing, revising, visualization.

**Faiz Tuma MD, MME, MDE, EdS** writing original draft, editing, revising, finalizing and supervising.

## Registration of research studies

This is a review study that involves no experiment or data to register.

## Guarantor

Aussama K. Nassar MD, MSc FACS, FRCSC.

## Declaration of competing interest

The authors report no conflicts of interest.
